# Association of low magnesium status with incident intracranial hemorrhage: a matched-control database study

**DOI:** 10.3389/fnut.2026.1886321

**Published:** 2026-08-06

**Authors:** Kuo-Chuan Hung, Ting-Sian Yu, Yi-Chen Lai, I-Wen Chen

**Affiliations:** 1 Department of Anesthesiology, Chi Mei Medical Center, Tainan, Taiwan; 2 Department of Anesthesiology, E-Da Hospital, I-Shou University, Kaohsiung, Taiwan; 3 Department of Anesthesiology, Chi Mei Hospital, Liouying, Tainan, Taiwan

**Keywords:** intracerebral hemorrhage, intracranial hemorrhage, magnesium, propensity score matching, retrospective cohort study, subarachnoid hemorrhage

## Abstract

**Background:**

Magnesium is involved in vascular tone, endothelial function, and coagulation pathways, yet evidence directly linking magnesium status to hemorrhagic cerebrovascular events remains limited. We examined whether chronic low serum magnesium levels are associated with incident nontraumatic intracranial hemorrhage in a large adult population.

**Methods:**

This retrospective cohort study used the TriNetX Global Collaborative Network (2010–2024). Low magnesium status was defined as two serum magnesium measurements <1.7 mg/dL within 1 year, and the comparator cohort had two normal values (1.70–2.20 mg/dL). A 3-month landmark design was applied, and 1:1 propensity score matching was performed for demographics, comorbidities, medications, and laboratory markers. The primary outcome was incident nontraumatic intracranial hemorrhage over 5 years. Secondary outcomes included intracerebral hemorrhage, subarachnoid hemorrhage, all-cause mortality, and systolic blood pressure >180 mmHg. Sensitivity, subgroup, and exposure gradient analyses were conducted.

**Results:**

After matching, 266,133 patients remained in each group. Low magnesium status was associated with a higher intracranial hemorrhage risk (HR, 1.74; 95% CI, 1.59–1.89; *p* < 0.001), as well as intracerebral hemorrhage (HR, 1.79; *p* < 0.001), subarachnoid hemorrhage (HR, 1.64; *p* < 0.001), all-cause mortality (HR, 1.49; *p* < 0.001), and systolic blood pressure >180 mmHg (HR, 1.07; *p* < 0.001). This association remained consistent across the sensitivity and subgroup analyses. Severe low magnesium levels (<1.5 mg/dL) showed a stronger association with intracranial hemorrhage (HR, 2.00; *p* < 0.001), suggesting an exposure-gradient relationship.

**Conclusion:**

Chronic low serum magnesium levels were associated with an increased risk of intracranial hemorrhage in this observational study. These findings warrant confirmation through prospective studies to clarify whether this association reflects a modifiable risk marker or the underlying illness severity.

## 1 Introduction

Nontraumatic intracranial hemorrhage, including intracerebral and subarachnoid hemorrhage, accounts for approximately 10–15% of all strokes but is disproportionately associated with mortality and long-term disability (1–4). Identifying clinically relevant and potentially modifiable risk markers remains clinically important because prevention strategies are still largely centered on blood pressure control and management of established vascular risks (5–7). Magnesium is an essential cation involved in vascular tone, endothelial function, blood pressure regulation, platelet aggregation, and coagulation pathways (8–11). Hypomagnesemia is common in clinical populations and has been linked to hypertension, diabetes, cardiovascular disease, and other adverse outcomes (12, 13). Meta-analyses of prospective cohort studies have reported inverse associations between higher dietary magnesium intake and the risks of total and ischemic stroke, whereas the associations with hemorrhagic stroke have been weaker or statistically non-significant (14–16). However, dietary estimates may not fully reflect clinically relevant magnesium depletion arising from impaired absorption, altered renal handling, medication exposure, systemic illnesses, or metabolic comorbidities. Accordingly, serum magnesium measurements may provide complementary information on clinically relevant magnesium status beyond dietary intake.

However, direct evidence linking magnesium status to hemorrhagic cerebrovascular events remains limited. Prior studies have mainly focused on prognosis after hemorrhagic stroke onset, including the associations between admission magnesium levels and hematoma volume, hematoma expansion, and clinical outcomes (17–19). Whether chronic low magnesium status precedes and is associated with subsequent incident intracranial hemorrhage in a large general adult population remains unclear. Therefore, we conducted a large-scale retrospective cohort study using the TriNetX Global Collaborative Network to examine the association between low serum magnesium status, defined as two confirmed low measurements within 1 year, and incident nontraumatic intracranial hemorrhage.

## 2 Methods

### 2.1 Data source and ethical statement

This study used data from the TriNetX Global Collaborative Network, a federated electronic health record research network that enables retrospective analyses across participating healthcare organizations while maintaining data de-identification at the source. The platform contains routinely collected clinical information, including patient demographics, diagnoses, procedures, medication records, laboratory results, and healthcare encounters. The TriNetX network has been widely used to support large-scale epidemiological and real-world evidence studies across diverse clinical fields (20–22). All analyses were performed within the TriNetX environment using aggregated, de-identified data; therefore, individual-level identifiers were not accessible to investigators. Because the study involved a secondary analysis of de-identified electronic health record data, informed consent was waived by the Institutional Review Board of Chi Mei Medical Center. This study was conducted in accordance with the Declaration of Helsinki and the applicable institutional requirements for retrospective observational research.

### 2.2 Inclusion and exclusion criteria

Adults aged ≥18 years with serum magnesium measurements recorded between January 1, 2010, and December 31, 2024, were eligible. Patients were required to have at least one healthcare visit within 5 years before the index date to ensure baseline observability. Low magnesium status was defined as two serum magnesium measurements <1.7 mg/dL within 1 year, and the second qualifying low magnesium value was assigned as the index date to reduce exposure misclassification from a single transient abnormal result. The comparator cohort included patients with two serum magnesium measurements within the normal range of 1.70–2.20 mg/dL within 1 year, with the second qualifying normal value assigned as the index date of the study. The cutoff of <1.7 mg/dL was selected according to the serum magnesium threshold available in the TriNetX laboratory data structure and was applied uniformly to all participating healthcare organizations within the network.

We excluded patients with a history of cerebrovascular disease before or near the index date, including cerebral infarction, transient ischemic attack, nontraumatic intracerebral hemorrhage, nontraumatic subarachnoid hemorrhage, and other or unspecified nontraumatic intracranial hemorrhage. Additional exclusions included end-stage renal disease, stage 4–5 chronic kidney disease (CKD), dialysis dependence, history of bariatric surgery, non-ruptured cerebral aneurysm, and non-ruptured cerebral artery dissection. To minimize confounding from acute illness–related magnesium abnormalities, patients with acute kidney failure, sepsis, severe sepsis, or critical care services within 1 month before the index date were excluded. The codes used to define the cohorts and exclusion criteria are summarized in Supplementary Table 1.

### 2.3 Landmark analysis

A 3-month landmark design was applied: patients who died or had an intracranial hemorrhage outcome within 90 days after the index date were excluded from the outcome analyses, and follow-up began on day 91. This approach was used to reduce reverse causation and early event bias, ensuring that hemorrhagic events were assessed after the stable classification of magnesium status. Patients were then followed up for up to 5 years for incident intracranial hemorrhage, including nontraumatic subarachnoid hemorrhage, nontraumatic intracerebral hemorrhage, and other or unspecified nontraumatic intracranial hemorrhage.

### 2.4 Data collection and propensity score matching

To reduce baseline differences between the low magnesium and reference cohorts, 1:1 propensity score matching was performed using a greedy nearest-neighbor algorithm. Propensity scores were estimated using prespecified clinically relevant covariates associated with magnesium status, intracranial hemorrhage risk, and healthcare utilization. These variables included age, sex, race, body mass index, hypertension, diabetes mellitus, ischemic heart disease, chronic kidney disease, heart failure, atrial fibrillation, peripheral vascular disease, liver disease, alcohol-related disorders, nicotine dependence, malnutrition, defined using ICD-10-CM codes for protein-calorie malnutrition and related malnutrition diagnoses (E40–E46), neoplasms, vitamin D deficiency, systemic connective tissue disorders, medication exposure, and laboratory markers reflecting renal function, inflammation, serum albumin, glycemic control, hematologic status, and blood pressure. Medication covariates included anticoagulants, platelet aggregation inhibitors, diuretics, magnesium supplementation, proton pump inhibitors, corticosteroids, antihypertensive agents, insulin, and non-insulin glucose-lowering drugs. Covariate balance before and after matching was assessed using standardized mean differences, with an absolute standardized mean difference <0.10 considered adequate balance. The overlap and redistribution of propensity scores before and after matching were further examined using density plots. Codes used to define these variables are summarized in Supplementary Table 2.

### 2.5 Primary and secondary outcomes

The primary outcome was incident nontraumatic intracranial hemorrhage, defined as nontraumatic intracerebral hemorrhage, nontraumatic subarachnoid hemorrhage, or other/unspecified nontraumatic intracranial hemorrhage, identified using ICD-10-CM codes I61, I60, and I62, respectively. Secondary outcomes included the individual hemorrhage subtypes, namely nontraumatic intracerebral hemorrhage (I61) and nontraumatic subarachnoid hemorrhage (I60), all-cause mortality, and systolic blood pressure >180 mmHg. All-cause mortality was identified using the TriNetX deceased status and ICD-10-CM code R99, and systolic blood pressure >180 mmHg was defined using the TriNetX laboratory/vital sign code TNX:9085. All outcomes were ascertained after a 3-month landmark period. Patients were followed up from the landmark date until the first occurrence of the outcome being evaluated, death, the last recorded encounter in the electronic health record, or the end of the 5-year follow-up period, whichever occurred first.

### 2.6 Positive and negative control outcomes

Prespecified control outcome analyses were performed to assess the credibility and specificity of the results. Incident ischemic stroke and hypocalcemia were selected as positive control outcomes because they are clinically plausible events associated with vascular risk (14–16) and magnesium-related electrolyte disturbances, respectively. Acute appendicitis (ICD-10-CM K35) was selected as a negative control outcome because no strong biological association with magnesium status was anticipated. These analyses were used to evaluate whether the observed associations were directionally consistent with biological plausibility, while also assessing potential residual confounding or nonspecific detection bias.

### 2.7 Sensitivity, subgroup, and additional analyses

Robustness was assessed using a prespecified sensitivity analysis. Model I excluded patients who died during the follow-up period. Model II was applied to a 1-year landmark period. Model III excluded patients with a history of malnutrition, hypoalbuminemia, and chronic kidney disease. An additional sensitivity analysis (Model IV) was performed by repeating propensity score matching with further inclusion of thrombocytopenia and other hemorrhagic/coagulation disorders (ICD-10-CM D65–D69), iron deficiency anemia (ICD-10-CM D50), and disorders of fluid, electrolyte, and acid–base balance (ICD-10-CM E87) as matching variables. Sex- and age-stratified subgroup analyses were conducted by repeating propensity score matching and outcome analyses within each stratum. Age groups were categorized as 18–65 and >65 years. Interaction testing was used to evaluate whether the association between low magnesium status and intracranial hemorrhage differed by sex or age.

To explore the exposure-gradient relationship, analyses were repeated among patients with severely low magnesium status, defined as two serum magnesium measurements <1.5 mg/dL within 1 year. The same index date definition, exclusion criteria, landmark period, propensity score matching approach, and outcome definitions were applied.

In addition, a high magnesium status was examined separately to assess whether the risk of hemorrhage followed a possible U-shaped pattern. High magnesium status was defined as two serum magnesium measurements >2.2 mg/dL within 1 year and was compared with the normal magnesium reference cohort using the same analytical framework.

### 2.8 Statistical analysis

Time-to-event analyses were conducted using Cox proportional hazards models, with results reported as hazard ratios (HR) and 95% confidence intervals (CI). Between-group differences in event-free survival were evaluated using log-rank tests, and the proportional hazards assumption was assessed using the Schoenfeld residuals. In addition to propensity score–matched analysis, multivariable Cox proportional hazards regression was performed in the overall cohort to further evaluate the association between magnesium status and incident intracranial hemorrhage after adjustment for demographic characteristics and comorbidities.

Because death may preclude subsequent diagnosis of intracranial hemorrhage, competing risk analyses were also performed using the Aalen–Johansen estimator, with intracranial hemorrhage and death treated as mutually exclusive outcomes. These analyses were descriptive and conducted separately within each cohort; therefore, no formal between-group subdistribution hazard ratios were estimated.

*E*-values were calculated for the primary outcome to estimate the minimum strength of association that an unmeasured confounder would need to have with both magnesium status and intracranial hemorrhage to fully explain the observed association. Statistical significance was defined as a two-sided *α* of 0.05 for the primary analyses. Secondary, control, sensitivity, subgroup, exposure-gradient, high-magnesium, and multivariable analyses were considered exploratory, and no multiplicity adjustments were applied to them. All analyses used the available data without imputation.

## 3 Results

### 3.1 Patient selection and baseline characteristics

**Figure 1:**
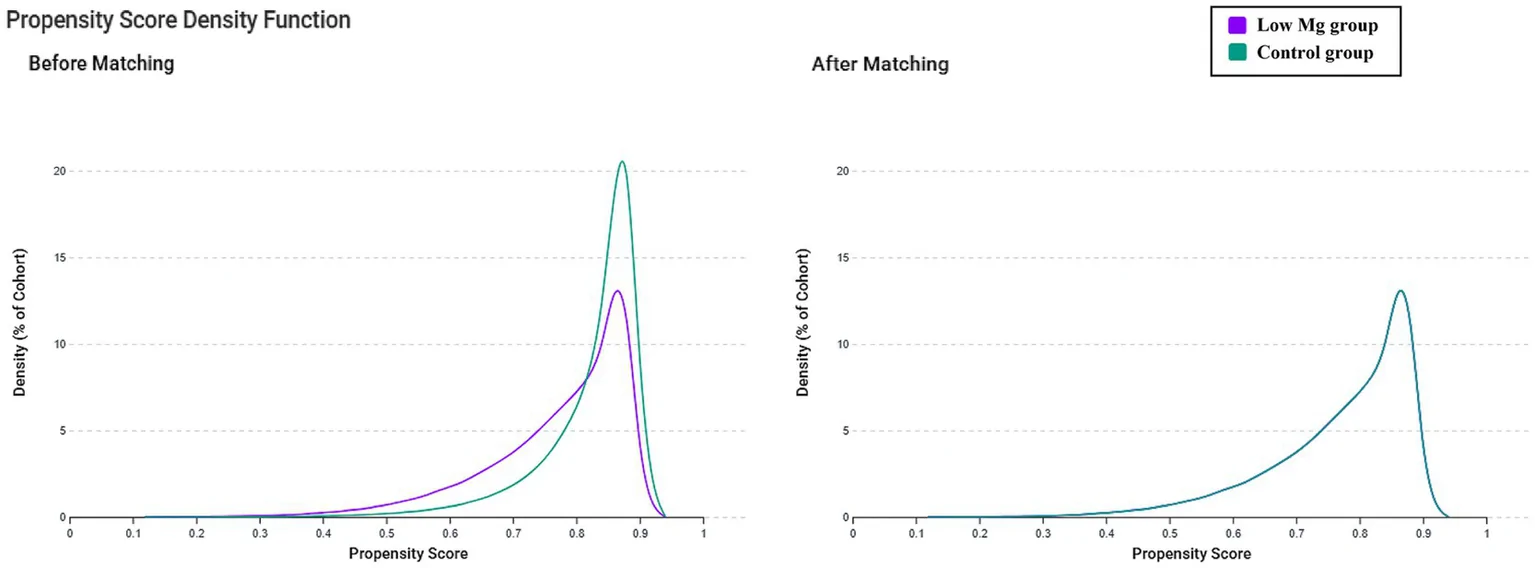
Propensity score density distributions before and after matching propensity score density distributions before and after 1:1 propensity score matching are shown for the low and normal magnesium cohorts. After matching, the distributions showed improved overlap between the cohorts, indicating an improved balance in the measured baseline characteristics.

A total of 266,272 patients with low magnesium status and 1,260,563 patients with normal magnesium status met the eligibility criteria (Table 1). After 1:1 propensity score matching, 266,133 patients remained in each group. Before matching, several covariates showed meaningful imbalances, such as hypertension, neoplasms, diabetes mellitus, anticoagulant use, and insulin use. After matching, all standardized mean differences were reduced to below 0.10, suggesting an adequate covariate balance for the measured baseline variables (Table 1). Propensity score density plots showed improved overlap between the cohorts after matching (Figure 1).

**Table 1:** Baseline characteristics of patients with low magnesium status and matched controls before and after propensity score matching.

Variables	Before matching			After matching		
	Low Mg group (*n* = 266,272)	Control group (*n* = 1,260,563)	SMD	Low Mg group (*n* = 266,133)	Control group (*n* = 266,133)	SMD
Patient characteristics						
Age at index (years)	57.8 ± 17.6	55.8 ± 18.9	0.107	57.8 ± 17.6	58.2 ± 17.7	0.026
BMI ≥ 30 (kg/m^2^)	81,314 (30.5)	356,952 (28.3)	0.049	81,279 (30.5)	78,231 (29.4)	0.025
Female	150,250 (56.4)	711,069 (56.4)	0.000	150,198 (56.4)	148,804 (55.9)	0.011
White	169,154 (63.5)	827,234 (65.6)	0.044	169,080 (63.5)	166,459 (62.5)	0.020
Black or African American	47,592 (17.9)	202,971 (16.1)	0.047	47,558 (17.9)	48,213 (18.1)	0.006
Asian	5,949 (2.2)	34,192 (2.7)	0.031	5,949 (2.2)	5,974 (2.2)	0.001
Comorbidities and healthcare utilization						
Factors influencing health status and contact with health services	157,626 (59.2)	671,948 (53.3)	0.119	157,502 (59.2)	151,698 (57.0)	0.044
Essential (primary) hypertension	107,164 (40.2)	400,331 (31.8)	0.178	107,047 (40.2)	103,277 (38.8)	0.029
Neoplasms	100,125 (37.6)	349,637 (27.7)	0.212	100,022 (37.6)	100,250 (37.7)	0.002
Disorders of lipoprotein metabolism and other lipidemias	76,300 (28.7)	314,542 (25.0)	0.084	76,246 (28.7)	72,097 (27.1)	0.035
Diabetes mellitus	65,406 (24.6)	168,669 (13.4)	0.288	65,287 (24.5)	64,717 (24.3)	0.005
Overweight and obesity	38,616 (14.5)	156,476 (12.4)	0.061	38,588 (14.5)	36,274 (13.6)	0.025
Ischemic heart diseases	36,725 (13.8)	134,296 (10.7)	0.096	36,684 (13.8)	35,196 (13.2)	0.016
Nicotine dependence	36,527 (13.7)	131,817 (10.5)	0.100	36,458 (13.7)	34,809 (13.1)	0.018
Diseases of liver	32,743 (12.3)	80,282 (6.4)	0.205	32,628 (12.3)	31,417 (11.8)	0.014
Disorders of thyroid gland	31,640 (11.9)	137,383 (10.9)	0.031	31,620 (11.9)	29,643 (11.1)	0.023
Vitamin D deficiency	22,161 (8.3)	103,654 (8.2)	0.004	22,145 (8.3)	20,280 (7.6)	0.026
Alcohol related disorders	20,927 (7.9)	48,019 (3.8)	0.173	20,829 (7.8)	20,349 (7.6)	0.007
Heart failure	20,340 (7.6)	66,997 (5.3)	0.095	20,310 (7.6)	19,741 (7.4)	0.008
Atrial fibrillation and flutter	19,308 (7.3)	77,008 (6.1)	0.046	19,295 (7.3)	18,158 (6.8)	0.017
Chronic kidney disease (CKD)	16,957 (6.4)	54,070 (4.3)	0.093	16,924 (6.4)	16,498 (6.2)	0.007
Malnutrition	11,386 (4.3)	21,579 (1.7)	0.151	11,307 (4.2)	10,329 (3.9)	0.019
Peripheral vascular diseases	9,759 (3.7)	31,704 (2.5)	0.066	9,742 (3.7)	9,348 (3.5)	0.008
Cerebrovascular diseases	8,134 (3.1)	28,520 (2.3)	0.049	8,117 (3.1)	7,745 (2.9)	0.008
Systemic connective tissue disorders	4,967 (1.9)	20,412 (1.6)	0.019	4,962 (1.9)	4,769 (1.8)	0.005
Laboratory data						
Hemoglobin ≥ 12 g/dL	173,012 (65.0)	747,115 (59.3)	0.118	172,889 (65.0)	173,359 (65.1)	0.004
Albumin ≥ 3.5 g/dL	158,135 (59.4)	628,926 (49.9)	0.192	158,011 (59.4)	157,479 (59.2)	0.004
HbA1c ≥ 9%	16,723 (6.3)	34,955 (2.8)	0.169	16,666 (6.3)	16,546 (6.2)	0.002
eGFR ≥ 60 mL/min/1.73 m^2^	183,139 (68.8)	763,254 (60.5)	0.173	183,004 (68.8)	183,998 (69.1)	0.008
C-reactive protein ≥ 10 mg/L	29,807 (11.2)	80,935 (6.4)	0.169	29,734 (11.2)	29,760 (11.2)	0.000
SBP > 180 mmHg	26,743 (10.0)	87,846 (7.0)	0.110	26,707 (10.0)	26,005 (9.8)	0.009
Medications						
Corticosteroids for systemic use	110,970 (41.7)	422,461 (33.5)	0.169	110,875 (41.7)	107,199 (40.3)	0.028
Anticoagulants	104,838 (39.4)	311,378 (24.7)	0.318	104,700 (39.3)	104,068 (39.1)	0.005
Proton pump inhibitors	89,069 (33.5)	282,183 (22.4)	0.249	88,944 (33.4)	86,188 (32.4)	0.022
Beta blockers/related	77,139 (29.0)	265,714 (21.1)	0.183	77,047 (29.0)	74,308 (27.9)	0.023
Diuretics	74,134 (27.8)	231,308 (18.4)	0.227	74,032 (27.8)	71,711 (26.9)	0.020
Magnesium supplementation	56,433 (21.2)	161,012 (12.8)	0.226	56,333 (21.2)	55,404 (20.8)	0.009
Platelet aggregation inhibitors	54,322 (20.4)	194,655 (15.4)	0.130	54,261 (20.4)	52,060 (19.6)	0.021
Insulins and analogues	53,377 (20.0)	118,695 (9.4)	0.303	53,252 (20.0)	52,016 (19.5)	0.012
Calcium channel blockers	48,137 (18.1)	166,065 (13.2)	0.135	48,082 (18.1)	46,115 (17.3)	0.019
Blood glucose lowering drugs, excl. Insulins	41,707 (15.7)	103,034 (8.2)	0.233	41,630 (15.6)	40,852 (15.4)	0.008
Antihypertensive combinations	1,440 (0.5)	5,012 (0.4)	0.021	1,439 (0.5)	1,403 (0.5)	0.002

Data are presented as *n* (%) or mean ± SD. An absolute standardized mean difference of <0.10 indicated adequate balance. BMI, body mass index; CKD, chronic kidney disease; CRP, C-reactive protein; eGFR, estimated glomerular filtration rate; HbA1c, hemoglobin A1c; Mg, magnesium; SBP, systolic blood pressure; SD, standard deviation; SMD, standardized mean difference.

### 3.2 Primary and secondary outcomes

During follow-up extending up to 5 years after the 3-month landmark period, low magnesium status was associated with a higher risk of intracranial hemorrhage than normal magnesium status (HR, 1.74; 95% CI, 1.59–1.89; *p* < 0.001) (Table 2). The *E*-value was 2.87 for the point estimate and 2.56 for the lower confidence limit, suggesting that an unmeasured confounder would need to be associated with both low magnesium status and intracranial hemorrhage by at least these magnitudes, beyond the measured covariates, to fully explain the observed association. Similar associations were observed for the individual hemorrhage subtypes, including intracerebral hemorrhage (HR, 1.79; *p* < 0.001) and subarachnoid hemorrhage (HR, 1.64; *p* < 0.001). Low magnesium status was also associated with higher all-cause mortality (HR, 1.49; *p* < 0.001) and a modestly higher risk of systolic blood pressure >180 mmHg (HR, 1.07; *p* < 0.001).

**Table 2:** Association between low magnesium status and 5-year clinical outcomes after propensity score matching.

Outcome	Low Mg group (*n* = 266,133)	Control group (*n* = 266,133)	HR (95% CI)	*p* value
	Events (%)	Events (%)		
Primary outcome				
Intracranial hemorrhage	1,388 (0.52)	824 (0.31)	1.74 (1.59–1.89)	<0.001
Secondary outcomes				
Intracerebral hemorrhage	737 (0.28)	425 (0.16)	1.79 (1.59–2.01)	<0.001
Subarachnoid hemorrhage	343 (0.13)	216 (0.08)	1.64 (1.38–1.94)	<0.001
Mortality	31,679 (11.90)	22,067 (8.29)	1.49 (1.46–1.51)	<0.001
SBP > 180 mmHg	38,663 (14.53)	37,178 (13.97)	1.07 (1.06–1.09)	<0.001
Positive control outcome				
Stroke	3,801 (1.43)	2,491 (0.94)	1.58 (1.50–1.66)	<0.001
Hypocalcemia	133,905 (50.32)	103,539 (38.91)	1.47 (1.46–1.49)	<0.001
Negative control outcome				
Acute appendicitis	994 (0.37)	1,021 (0.38)	1.00 (0.91–1.09)	0.922

CI, confidence interval; HR, hazard ratio; SBP, systolic blood pressure. Intracranial hemorrhage was defined as nontraumatic intracerebral hemorrhage, nontraumatic subarachnoid hemorrhage, or other/unspecified nontraumatic intracranial hemorrhage.

The positive control analyses were directionally consistent with biological plausibility, as low magnesium status was associated with a higher risk of ischemic stroke (HR, 1.58; p < 0.001) and hypocalcemia (HR, 1.47; p < 0.001) (Table 2). In contrast, the negative control outcome, acute appendicitis, was not associated with magnesium status (HR, 1.00; *p* = 0.922), providing partial reassurance against nonspecific detection or healthcare-utilization bias. Nevertheless, these control outcome analyses cannot exclude residual confounding or ascertainment biases.

### 3.3 Sensitivity and subgroup analyses

The association between low magnesium status and intracranial hemorrhage was generally consistent across all prespecified sensitivity analyses (Table 3). After excluding patients who died during follow-up (Model I), the point estimate was attenuated but remained significant (HR 1.62, *p* < 0.001). The association persisted when a 1-year landmark period was applied (Model II; HR 1.72, *p* < 0.001) and was preserved after excluding those with malnutrition, hypoalbuminemia, or chronic kidney disease (Model III; HR 1.74, *p* < 0.001). In Model IV, which additionally matched hematologic, coagulation, iron-status, and electrolyte disorders, the association with intracranial hemorrhage remained significant but was attenuated (HR 1.57, *p* < 0.001). Similar patterns were observed for intracerebral and subarachnoid hemorrhage subtypes across all sensitivity models.

**Table 3:** Sensitivity analyses for the association between low magnesium status and 5-year clinical outcomes.

Outcome	Model I (*n* = 206,349)		Model II (*n* = 245,934)		Model III (*n* = 76,534)		Model IV (*n* = 235,436)	
	HR (95% CI)	*p*-value	HR (95% CI)	*p*-value	HR (95% CI)	*p*-value	HR (95% CI)	*p*-value
Intracranial hemorrhage	1.62 (1.44–1.82)	<0.001	1.72 (1.53–1.92)	<0.001	1.74 (1.46–2.07)	<0.001	1.57 (1.44–1.72)	<0.001
Intracerebral hemorrhage	1.56 (1.33–1.83)	<0.001	1.68 (1.43–1.96)	<0.001	1.71 (1.33–2.19)	<0.001	1.62 (1.43–1.84)	<0.001
Subarachnoid hemorrhage	1.48 (1.19–1.85)	0.001	1.63 (1.31–2.02)	<0.001	1.88 (1.32–2.67)	<0.001	1.54 (1.29–1.83)	<0.001
Mortality	—	—	1.54 (1.50–1.58)	<0.001	1.62 (1.55–1.68)	<0.001	1.42 (1.39–1.45)	<0.001
SBP > 180 mmHg	1.06 (1.04–1.08)	<0.001	1.05 (1.04–1.07)	<0.001	1.10 (1.07–1.13)	<0.001	1.05 (1.03–1.07)	<0.001

Model I excluded patients who died during follow-up. Model II applied a 1-year landmark period. Model III excluded patients with a history of malnutrition, defined using ICD-10-CM codes for protein-calorie malnutrition and related malnutrition diagnoses (E40–E46), hypoalbuminemia, or chronic kidney disease. Model IV repeated propensity score matching after additionally incorporating thrombocytopenia, coagulation defects/purpura/other hemorrhagic conditions, iron deficiency anemia, and disorders of fluid, electrolyte, and acid–base balance as matching variables. CI, confidence interval; HR, hazard ratio; ICH, intracerebral hemorrhage; Mg, magnesium; SBP, systolic blood pressure.

In sex-stratified analyses, the association between low magnesium status and intracranial hemorrhage was similar between males (HR 1.76, *p* < 0.001) and females (HR 1.64, *p* < 0.001), with no significant interaction (*p* = 0.423) (Table 4). Age-stratified analyses also showed comparable associations in both the 18–65 (HR 1.68, *p* < 0.001) and > 65 years (HR 1.71, *p* < 0.001) groups, without significant interaction (*p* = 0.849), except for mortality, where the association was stronger in the younger subgroup (P for interaction < 0.001) (Table 5).

**Table 4:** Sex-stratified analyses of low magnesium status and 5-year clinical outcomes.

Outcomes	Male (*n* = 114,461 for each group)		Female (*n* = 150,222 for each group)		P for interaction
	HR (95% CI)	*p* value	HR (95% CI)	*p* value	
Intracranial hemorrhage	1.76 (1.56–1.99)	<0.001	1.64 (1.45–1.85)	<0.001	0.423
Intracerebral hemorrhage	1.68 (1.42–1.98)	<0.001	1.75 (1.48–2.07)	<0.001	0.736
Subarachnoid hemorrhage	1.78 (1.38–2.29)	<0.001	1.47 (1.17–1.85)	0.001	0.285
Mortality	1.47 (1.44–1.51)	<0.001	1.50 (1.47–1.54)	<0.001	0.235
SBP > 180 mmHg	1.07 (1.05–1.10)	<0.001	1.06 (1.04–1.08)	<0.001	0.540

HR, hazard ratio; CI, confidence interval; SBP, systolic blood pressure.

**Table 5:** Age-stratified analyses of low magnesium status and 5-year clinical outcomes.

Outcomes	18–65 years (*n* = 116,940 for each group)		>65 years (*n* = 149,054 for each group)		P for interaction
	HR (95% CI)	*p* value	HR (95% CI)	*p* value	
Intracranial hemorrhage	1.68 (1.45–1.95)	<0.001	1.71 (1.54–1.90)	<0.001	0.849
Intracerebral hemorrhage	1.69 (1.38–2.07)	<0.001	1.76 (1.52–2.03)	<0.001	0.749
Subarachnoid hemorrhage	1.47 (1.08–2.00)	0.013	1.68 (1.37–2.06)	<0.001	0.474
Mortality	1.70 (1.64–1.76)	<0.001	1.49 (1.46–1.52)	<0.001	<0.001
SBP > 180 mmHg	1.09 (1.06–1.12)	<0.001	1.08 (1.06–1.10)	<0.001	0.587

HR, hazard ratio; CI, confidence interval; SBP, systolic blood pressure.

### 3.4 Multivariable Cox regression and competing-risk analysis

In the multivariable Cox regression model adjusted for demographic characteristics, comorbidities, and history of cerebrovascular disease, low magnesium status remained associated with incident intracranial hemorrhage (HR 1.81, 95% CI 1.70–1.93; *p* < 0.001) (Table 6). Among other covariates, alcohol-related disorders (HR 1.44, *p* < 0.001), cerebrovascular diseases (HR 1.51, *p* < 0.001), nicotine dependence (HR 1.31, *p* < 0.001), and malnutrition (HR 1.32, *p* < 0.001) showed relatively larger associations with intracranial hemorrhage risk.

**Table 6:** Multivariable Cox regression analysis for incident intracranial hemorrhage.

Variables	HR (95% CI)	*p* value
Low Mg status vs. normal Mg status	1.81 (1.70–1.93)	<0.001
Male	1.28 (1.21–1.36)	<0.001
Age at index	1.03 (1.03–1.03)	<0.001
Essential hypertension	1.01 (0.94–1.08)	0.807
Neoplasms	1.23 (1.16–1.31)	<0.001
Diabetes mellitus	1.08 (1.00–1.16)	0.059
Ischemic heart diseases	0.98 (0.90–1.06)	0.574
Nicotine dependence	1.31 (1.20–1.43)	<0.001
Diseases of liver	1.24 (1.13–1.36)	<0.001
Alcohol-related disorders	1.44 (1.27–1.62)	<0.001
Heart failure	1.23 (1.11–1.36)	<0.001
Atrial fibrillation and flutter	1.19 (1.09–1.31)	<0.001
Chronic kidney disease	1.21 (1.09–1.34)	<0.001
Malnutrition	1.32 (1.12–1.56)	0.001
Cerebrovascular diseases	1.51 (1.33–1.71)	<0.001
White	0.95 (0.90–1.02)	0.135

CI, confidence interval; HR, hazard ratio; Mg, magnesium.

Competing risk analysis using the Aalen–Johansen estimator showed that the 5-year cumulative incidence of intracranial hemorrhage was higher in the low magnesium cohort (0.75%) than in the control cohort (0.40%), whereas the cumulative incidence of death was also higher in the low magnesium cohort (16.31% vs. 8.93%) (Table 7). These descriptive results suggest that the observed association was not solely attributable to the differential mortality between groups.

**Table 7:** Competing-risk analysis for intracranial hemorrhage and death during 5-year follow-up.

Cohort	Event type	Patient count	% of cohort	Aalen–Johansen cumulative incidence at 5 years (%)
Control group (*n* = 1,260,563)	Intracranial hemorrhage	3,584	0.27	0.40
Low Mg group (*n* = 266,272)	Intracranial hemorrhage	1,409	0.52	0.75
Control group (*n* = 1,260,563)	Death	83,785	6.36	8.93
Low Mg group (*n* = 266,272)	Death	32,098	11.73	16.31

Competing risk analyses were performed using the Aalen–Johansen estimator, with intracranial hemorrhage and death treated as mutually exclusive outcomes. Analyses were descriptive and generated separately within each cohort; therefore, no formal between-group subdistribution hazard ratios were estimated. Mg, magnesium.

### 3.5 Exposure-gradient and high-magnesium analyses

In the exposure-gradient analysis, severely low magnesium status (serum magnesium <1.5 mg/dL) was associated with a stronger risk of intracranial hemorrhage (HR 2.00, 95% CI 1.80–2.21; *p* < 0.001) compared with the primary analysis, with similar patterns for intracerebral hemorrhage (HR 2.01, *p* < 0.001) and subarachnoid hemorrhage (HR 1.61, *p* < 0.001) (Table 8). This pattern suggests an exposure-gradient association between low magnesium levels and intracranial hemorrhage risk.

**Table 8:** Exposure-gradient analysis of severe low magnesium status and 5-year clinical outcomes.

Outcome	Severe low Mg group (*n* = 169,943)	Control group (*n* = 169,943)	HR (95% CI)	*p* value
	Events (%)	Events (%)		
Intracranial hemorrhage	1,062 (0.63)	552 (0.33)	2.00 (1.80–2.21)	<0.001
Intracerebral hemorrhage	550 (0.32)	284 (0.17)	2.01 (1.74–2.32)	<0.001
Subarachnoid hemorrhage	247 (0.15)	159 (0.09)	1.61 (1.32–1.97)	<0.001
Mortality	22,201 (13.06)	14,758 (8.68)	1.57 (1.54–1.60)	<0.001
SBP > 180 mmHg	24,827 (14.61)	24,276 (14.29)	1.06 (1.04–1.08)	<0.001
Stroke	2,698 (1.59)	1,543 (0.91)	1.82 (1.71–1.94)	<0.001
Hypocalcemia	89,519 (52.68)	67,242 (39.57)	1.55 (1.54–1.57)	<0.001
Acute appendicitis	630 (0.37)	666 (0.39)	0.97 (0.87–1.08)	0.615

HR, hazard ratio; ICH, intracerebral hemorrhage; Mg, magnesium; SBP, systolic blood pressure.

In a separate exploratory analysis, high magnesium status (>2.2 mg/dL) was also associated with an increased intracranial hemorrhage risk compared with the normal reference cohort (HR 1.85, 95% CI 1.68–2.03; *p* < 0.001) (Table 9). Notably, high magnesium status was not associated with elevated systolic blood pressure (HR 1.00, *p* = 0.675), in contrast to the low magnesium status. Because this analysis was exploratory and conducted as a separate matched comparison, these findings should be interpreted as hypothesis-generating and should not be considered evidence confirming a U-shaped association.

**Table 9:** Association between high magnesium status and 5-year clinical outcomes.

Outcome	High Mg group (*n* = 198,800)	Control group (*n* = 198,800)	HR (95% CI)	*p* value
	Events (%)	Events (%)		
Intracranial hemorrhage	1,173 (0.59)	638 (0.32)	1.85 (1.68–2.03)	<0.001
Intracerebral hemorrhage	611 (0.31)	348 (0.18)	1.76 (1.54–2.01)	<0.001
Subarachnoid hemorrhage	282 (0.14)	168 (0.08)	1.68 (1.39–2.04)	<0.001
Mortality	23,339 (11.74)	16,737 (8.42)	1.41 (1.38–1.44)	<0.001
SBP > 180 mmHg	24,618 (12.38)	24,670 (12.41)	1.00 (0.99–1.02)	0.675

CI, confidence interval; HR, hazard ratio; high magnesium status was defined as two serum magnesium measurements >2.20 mg/dL within 1 year. CI, confidence interval; HR, hazard ratio; Mg, magnesium; SBP, systolic blood pressure.

## 4 Discussion

This large-scale retrospective cohort study found that chronic low serum magnesium levels were associated with a higher incidence of intracranial hemorrhage in the general adult population. The association appeared stronger among patients with severe low magnesium status, and the separate high-magnesium analysis suggested that both low and high serum magnesium levels may be associated with an increased hemorrhagic risk. Because serum magnesium can be obtained through a readily available clinical blood test, these findings suggest that repeated low serum magnesium levels may help characterize patients with a higher hemorrhagic cerebrovascular risk, although causality cannot be established from the present observational design.

Prior meta-analyses of prospective cohort studies have consistently reported inverse associations between dietary magnesium intake and ischemic stroke risk (14–16), whereas associations with hemorrhagic stroke subtypes have been absent or non-significant. Our findings differ from those of prior dietary-based studies by identifying an association when magnesium exposure was defined using repeated serum magnesium measurements rather than estimated dietary intake. This discrepancy may reflect differences between dietary intake estimates and measured serum magnesium status because dietary questionnaires may not capture magnesium depletion related to impaired absorption, renal wasting, medication exposure, systemic illness, or metabolic comorbidity. However, an alternative explanation is residual confounding related to clinical conditions associated with low magnesium status. Because both the low magnesium and reference cohorts were required to have two serum magnesium measurements within 1 year, differential magnesium testing itself is unlikely to fully explain the observed association. Nevertheless, low magnesium values may partly reflect a greater illness burden, nutritional vulnerability, medication exposure, renal or gastrointestinal magnesium handling, or systemic inflammation rather than a direct biological effect. In the current study, our sensitivity analysis excluding patients with malnutrition, hypoalbuminemia, or chronic kidney disease was designed to partly address this concern and yielded a comparable risk.

Hypomagnesemia is associated with endothelial dysfunction, increased vascular tone, and impaired blood pressure regulation (8–11). In our study, low magnesium levels were associated with a modestly elevated risk of systolic blood pressure exceeding 180 mmHg (HR, 1.07), consistent with the findings of the CRIC Study, in which higher serum magnesium levels were associated with lower systolic and diastolic blood pressures and a lower risk of hypertension among patients with chronic kidney disease (23). However, the modest association with systolic blood pressure >180 mmHg suggests that severe hypertension alone is unlikely to fully explain the relationship between low magnesium status and intracranial hemorrhage. Beyond hemodynamic pathways, prior studies have linked magnesium deficiency to coagulation disturbances in critical illness (9, 24) and to oxidative stress, pro-inflammatory endothelial signaling, and impaired endothelial barrier dysfunction (25–28). These mechanisms provide biological plausibility but do not establish causality in the present study. Thus, a low magnesium status may reflect a broader phenotype of vascular dysregulation, impaired hemostatic balance, and systemic illness rather than a single isolated pathway.

The exposure-gradient analysis showed a stronger association with severe hypomagnesemia, defined as serum magnesium <1.5 mg/dL, than with the primary low magnesium definition. This pattern was observed for both intracranial hemorrhage (HR, 2.00 vs. 1.74) and intracerebral hemorrhages (HR, 2.01 vs. 1.79). In contrast, the gradient was less evident for subarachnoid hemorrhage, which may reflect the differences in the underlying biology of parenchymal and aneurysmal hemorrhages. The positive and negative control outcomes also supported the internal consistency of these findings. The association with ischemic stroke was directionally consistent with the Atherosclerosis Risk in Communities (ARIC) Study (29), which reported an inverse association between serum magnesium levels and incident ischemic stroke. By contrast, the null association with acute appendicitis (HR, 1.00) provides some reassurance that the observed cerebrovascular associations were unlikely to be explained solely by broad nonspecific detection or healthcare-utilization bias. Although the association persisted after multivariable adjustment, it should be interpreted as an association rather than evidence of causality, because unmeasured factors such as nutritional status, medication exposure, renal handling of magnesium, and severity of underlying illness may still contribute to the observed relationship.

A noteworthy finding was the possible U-shaped association. High magnesium status (>2.2 mg/dL) was also associated with an elevated intracranial hemorrhage risk (HR 1.85), comparable to the association with low magnesium levels. Similar nonlinear relationships have been reported in other populations with cardiovascular and kidney diseases. In acute myocardial infarction, serum magnesium showed a U-shaped association with in-hospital mortality and malignant arrhythmias, with the lowest risk at approximately 1.7–1.9 mg/dL (30). In CKD cohorts, abnormal magnesium levels have also been associated with adverse cardiovascular and mortality outcomes, suggesting that both deficient and excessive magnesium levels may have prognostic significance (31). Importantly, high magnesium levels showed no association with elevated systolic blood pressure (HR 1.00, *p* = 0.675), in contrast to low magnesium status, suggesting that the clinical profiles or pathways associated with hemorrhage risk may differ between low and high magnesium levels. Hypermagnesemia frequently reflects impaired renal clearance or severe comorbidities and may function as a marker of disease severity rather than a direct causative factor. The low- and high-magnesium analyses were conducted as separate propensity score–matched comparisons against the normal reference cohort. In addition, the TriNetX analytical environment does not provide patient-level data for continuous exposure modeling; therefore, we could not perform restricted cubic spline analysis or formally test nonlinearity. Accordingly, the observed exposure-gradient and high-magnesium findings should be interpreted as exploratory and hypothesis-generating, rather than as confirmation of a U-shaped or continuous dose–response association.

Several additional analyses were consistent with the primary results. Competing risk analysis using the Aalen–Johansen estimator showed that the 5-year cumulative incidence of intracranial hemorrhage remained higher in the low magnesium cohort (0.75% vs. 0.40%) despite a substantially higher cumulative incidence of death (16.31% vs. 8.93%), suggesting that the association was unlikely to be explained entirely by differential mortality. A formal subdistribution hazard ratio was not estimated; therefore, these competing risk results should be interpreted as descriptive and supportive. This association persisted even after excluding patients who died during follow-up (HR 1.62), although the attenuation may partly reflect the exclusion of terminal hemorrhagic events. Extending the landmark period to 1 year (HR 1.72) and excluding patients with malnutrition, hypoalbuminemia, or CKD (HR 1.74) yielded comparable results.

The clinical implications of these findings should be interpreted in light of the low absolute event rates. Although low magnesium status was associated with a substantial relative increase in intracranial hemorrhage risk, the absolute event rates remained low (0.52% vs. 0.31%). Therefore, low magnesium status should not be viewed as a standalone predictor or as evidence supporting magnesium-targeted intervention. The matching strategy was intended to reduce confounding and estimate the independent association between magnesium status and intracranial hemorrhage, rather than to establish serum magnesium as a standalone prediction tool. Rather, repeated low serum magnesium may represent a readily available risk marker that helps characterize a higher-risk clinical phenotype when interpreted together with established hemorrhagic risk factors. Prospective studies are needed to determine whether correction of magnesium abnormalities can modify intracranial hemorrhage risk.

Several important limitations of this study should be acknowledged. First, because this was an observational study, the findings should be interpreted as associations rather than as evidence of causality. Serum magnesium should be regarded as an imperfect surrogate for total body magnesium status. Although repeated serum magnesium measurements may reduce exposure misclassification from a single transient abnormal value, serum magnesium primarily reflects extracellular magnesium and may not fully capture intracellular magnesium concentration, total body magnesium stores, or the ionized, biologically active fraction within the neurovascular microenvironment. Serum magnesium levels may fluctuate over time and may be affected by acute illness, renal dysfunction, intravenous fluids, medications, nutritional status, gastrointestinal losses, glucose-related shifts, and systemic inflammation. Although we required two qualifying magnesium measurements and excluded recent acute illness, residual exposure misclassification remains possible. We did not perform a temporally proximal analysis because our primary objective was to evaluate baseline chronic low magnesium status rather than acute pre-event magnesium changes. Second, despite propensity score matching and multivariable adjustment, residual confounding cannot be excluded from the results. Potential unmeasured factors, including dietary magnesium intake, nutritional patterns, socioeconomic status, medication adherence, anticoagulant dose or intensity, blood pressure variability, frailty, and genetic susceptibility, may have influenced both magnesium status and hemorrhagic risk. Although patients with non-ruptured cerebral aneurysm and non-ruptured cerebral artery dissection were excluded at baseline, other structural intracranial vascular lesions, including arteriovenous malformation, cavernous malformation, and dural arteriovenous fistula, were not included in the predefined exclusion criteria. Therefore, residual confounding from these lesions cannot be completely excluded. Third, outcomes were identified using ICD codes without direct radiographic adjudication; therefore, outcome misclassification and the inability to distinguish hemorrhage mechanisms, such as hypertensive arteriopathy versus cerebral amyloid angiopathy, remain possible. Fourth, requiring two qualifying magnesium measurements within 1 year may introduce selection and survivorship bias because patients had to remain alive and engaged in healthcare long enough to undergo repeat testing. Finally, because the TriNetX Global Collaborative Network is predominantly based on participating healthcare organizations in the United States, generalizability to other healthcare systems, ethnic groups, and practice settings may be limited to some extent.

## 5 Conclusion

Chronic low serum magnesium status was associated with a higher risk of incident intracranial hemorrhage, with a stronger association observed among patients with severely low magnesium status and a possible U-shaped pattern across magnesium levels. These findings extend prior dietary-based evidence by suggesting that repeated serum magnesium measurements may offer complementary information for characterizing the risk of hemorrhagic cerebrovascular disease. Further prospective studies are needed to determine whether magnesium status is a modifiable contributor to intracranial hemorrhage risk or primarily reflects underlying illness severity.

## Data Availability

The datasets presented in this article are not readily available because The TriNetX data used in this study are subject to contractual restrictions and are not publicly available from the authors. Access is limited to authorized users through the TriNetX platform under an institutional data-use agreement. The investigators accessed only aggregated, de-identified results and did not have access to individual-level identifiers or raw patient-level data. Requests to access the datasets should be directed to https://live.trinetx.com.
