# Supplementary material for: Association of low magnesium status with incident intracranial hemorrhage: a matched-control database study
Source: Front Nutr. 2026 Aug 6;13:1886321. doi: 10.3389/fnut.2026.1886321 (PMC13489897; doi:10.3389/fnut.2026.1886321)
Supplement: Supplementary file 1 [file Table_1.docx]

**Supplemental Table 1.** Codes used for cohort definition and exclusions

| Category | Criterion | Code system | Code | Definition |
| --- | --- | --- | --- | --- |
| Inclusion | Age ≥18 years | Demographics | Age | Adult patients |
| Exposure | Low Mg | LOINC | 19123-9 | Serum Mg ≤1.69 mg/dL, twice within 1 year |
| Comparator | Normal Mg | LOINC | 19123-9 | Serum Mg 1.70–2.20 mg/dL, twice within 1 year |
| Baseline observability | Healthcare visit | TriNetX | TNX:Visit | Visit within 5 years before index date |
| Prior cerebrovascular disease | Ischemic stroke | ICD-10-CM | I63 | Cerebral infarction |
| Prior cerebrovascular disease | TIA | ICD-10-CM | G45 | Transient ischemic attack |
| Prior ICH / outcome exclusion | SAH | ICD-10-CM | I60 | Nontraumatic subarachnoid hemorrhage |
| Prior ICH / outcome exclusion | ICH | ICD-10-CM | I61 | Nontraumatic intracerebral hemorrhage |
| Prior ICH / outcome exclusion | Other intracranial hemorrhage | ICD-10-CM | I62 | Other/unspecified nontraumatic intracranial hemorrhage |
| Acute illness exclusion | Acute kidney failure | ICD-10-CM | N17 | Excluded within 1 month before index date |
| Acute illness exclusion | Sepsis | ICD-10-CM | A41, R65.2 | Sepsis or severe sepsis |
| Acute illness exclusion | Critical care | CPT | 1013729 | Critical care services |
| Renal exclusion | Advanced CKD / ESRD | ICD-10-CM | N18.4, N18.5, N18.6 | CKD stage 4–5 or ESRD |
| Renal exclusion | Dialysis dependence | ICD-10-CM | Z99.2 | Dependence on renal dialysis |
| Other exclusion | Bariatric surgery | ICD-10-CM / CPT | Z98.84; 1007385 | Bariatric surgery status/procedure |
| Other exclusion | Cerebral vascular lesion | ICD-10-CM | I67.0, I67.1 | Nonruptured dissection or aneurysm |
| Landmark exclusion | Death within 90 days | Demographics / ICD-10-CM | Deceased; R99 | Excluded during 3-month landmark period |

**Supplementary Table 2**. Codes used to define variables for propensity score matching

| Variable | Code / TriNetX term | Definition / threshold |
| --- | --- | --- |
| Age | Age at index | Age at cohort index date |
| Sex | F | Female sex |
| White race | Mar-06 | White |
| Black or African American race | May-54 | Black or African American |
| Asian race | Sep-28 | Asian |
| Essential hypertension | ICD-10-CM I10 | Essential primary hypertension |
| Overweight and obesity | ICD-10-CM E66 | Overweight and obesity |
| Diabetes mellitus | ICD-10-CM E08–E13 | Diabetes mellitus |
| Nicotine dependence | ICD-10-CM F17 | Nicotine dependence |
| Ischemic heart disease | ICD-10-CM I20–I25 | Ischemic heart diseases |
| Chronic kidney disease | ICD-10-CM N18 | Chronic kidney disease |
| Alcohol-related disorders | ICD-10-CM F10 | Alcohol-related disorders |
| Cerebrovascular diseases | ICD-10-CM I60–I69 | Cerebrovascular diseases |
| Malnutrition | ICD-10-CM E40–E46 | Malnutrition |
| Healthcare utilization / contact with health services | ICD-10-CM Z00–Z99 | Factors influencing health status and contact with health services |
| Heart failure | ICD-10-CM I50 | Heart failure |
| Atrial fibrillation and flutter | ICD-10-CM I48 | Atrial fibrillation and flutter |
| Systemic connective tissue disorders | ICD-10-CM M30–M36 | Systemic connective tissue disorders |
| Liver disease | ICD-10-CM K70–K77 | Diseases of liver |
| Thyroid disorders | ICD-10-CM E00–E07 | Disorders of thyroid gland |
| Vitamin D deficiency | ICD-10-CM E55 | Vitamin D deficiency |
| Neoplasms | ICD-10-CM C00–D49 | Neoplasms |
| Dyslipidemia | ICD-10-CM E78 | Disorders of lipoprotein metabolism and other lipidemias |
| Peripheral vascular disease | ICD-10-CM I73 | Other peripheral vascular diseases |
| Insulins and analogues | ATC A10A | Insulins and analogues |
| Non-insulin glucose-lowering drugs | ATC A10B | Blood glucose-lowering drugs, excluding insulins |
| Diuretics | CV700 | Diuretics |
| Magnesium supplementation | TN460 | Magnesium |
| Proton pump inhibitors | ATC A02BC | Proton pump inhibitors |
| Systemic corticosteroids | ATC H02 | Corticosteroids for systemic use |
| Anticoagulants | BL110 | Anticoagulants |
| Platelet aggregation inhibitors | BL117 | Platelet aggregation inhibitors |
| Beta blockers / related agents | CV100 | Beta blockers / related agents |
| Calcium channel blockers | CV200 | Calcium channel blockers |
| Antihypertensive combinations | CV400 | Antihypertensive combinations |
| Albumin | TNX:9045 | Albumin ≥3.5 g/dL |
| Estimated glomerular filtration rate | LOINC 98979-8 | eGFR ≥60 mL/min/1.73 m², CKD-EPI 2021 |
| Hemoglobin A1c | TNX:9037 | HbA1c ≥9% |
| C-reactive protein | TNX:9063 | CRP ≥10 mg/L |
| Hemoglobin | TNX:9014 | Hemoglobin ≥12 g/dL |
| Body mass index | TNX:9083 | BMI ≥30 kg/m² |
| Systolic blood pressure | TNX:9085 | Systolic blood pressure ≥180 mmHg |
